# Multilink Operation in IEEE 802.11be Wireless LANs: Backoff Overflow Problem and Solutions

**DOI:** 10.3390/s22093501

**Published:** 2022-05-04

**Authors:** Wisnu Murti, Ji-Hoon Yun

**Affiliations:** Department of Electrical and Information Engineering, Seoul National University of Science and Technology, Seoul 01811, Korea; wisnumurti@seoultech.ac.kr

**Keywords:** IEEE 802.11be, multilink operation, channel access, backoff, coexistence

## Abstract

The next-generation wireless LAN standard named IEEE 802.11be supports a multilink operation to cost-efficiently boost throughput performance, for which an efficient multilink channel scheme is essential. The synchronous channel access scheme with an enhancement allowing multilink transmission before backoff completion greatly enhances the performance of multilink devices with no simultaneous transmit and receive capability, for which, however, backoff count compensation is necessary for coexistence with legacy and other multilink devices. In this paper, we identify the backoff count overflow problem of the enhanced synchronous channel access scheme with backoff compensation, which becomes aggravated once triggered due to repeated compensations. Then, we propose four solutions to mitigate this problem: limiting consecutive free-riding transmissions, limiting a compensated backoff value, using the contention window value of a main link, and balancing transmissions between links. Through comparative evaluation and analyses for dense single-spot and indoor random deployment scenarios, we demonstrate in terms of throughput and latency that the proposed solutions successfully mitigate the problem while preserving the coexistence performance.

## 1. Introduction

A multilink operation (MLO) is a salient feature of the upcoming next-generation wireless LAN (WLAN) standard, i.e., IEEE 802.11be (also called extremely high throughput (EHT)) [1,2], that enables the simultaneous utilization of multiple links using individual frequency channels for both transmission and reception. The benefit of MLO is the simultaneous utilization of multiple frequency bands at a lower hardware cost than the approach of using a single multiband radio. Designing an efficient channel access operation for the presence of radio frequency (RF) power leakage between links to best exploit the benefit of MLO is still a challenge. In the IEEE 802.11be working group (WG), it was agreed that the multiple links involved in MLO may or may not have RF power leakage between them. A multilink device (MLD), which has links with power leakage between them, is unable to simultaneously transmit and receive (STR) across different links, as the power leakage from one link will make the other links unable to sense each’s channel medium. This type of MLD is called a non-STR MLD. On the other side, an MLD with no RF power leakage between its links will be able to simultaneously transmit and receive signals and thus is called an STR MLD.

The channel access scheme of IEEE 802.11be is classified into an asynchronous operation (Async) and a synchronous operation (Sync). In Async, each of the links belonging to an MLD independently performs a channel access process (backoff procedure, BO). Therefore, different links finish their BOs at different times; thus, their transmissions will be asynchronous between them, which is ideal for STR MLDs. In Sync, all links also perform individual BOs. When a link finishes its BO earlier than other links, it waits until the other links also finish their BOs. If the waiting link sees its channel busy, the link must rerun its BO. Because only simultaneous transmission initiation is allowed on all links, the RF power leakage between links does not affect the operation of Sync. However, Sync does not utilize multiple channels better than Async, since it requires all links to have zero backoff counts for transmission.

There has been a notable discussion on the enhancements of Sync for better multilink exploitation and a hybrid of the Sync and Async schemes for faster transmission (FT), called Sync-FT [3]. In this work, the coexistence issue of Sync-FT was discussed since legacy single-link devices (SLDs) may coexist with EHT MLDs in practical WLANs, and multiple solutions were proposed. This is because, with Sync-FT, a link of the MLD can transmit before its BO completion, which is called free-riding. This behavior prioritizes channel access for the MLD, resulting in long and frequent wait times for the legacy device. It was shown in the work that compensating the backoff count for each free-riding transmission achieved the best coexistence performance while having a marginal throughput decrease in MLDs. However, there may arise a backoff count overflow problem where the accumulated backoff count of an MLD resulting from repeated compensations becomes too large; thus, the MLD is not able to obtain a transmission opportunity by its own BO completion once an overflow occurs.

This paper aims to identify the backoff count overflow problem of the enhanced multilink channel access scheme (Sync-FT) with backoff compensation in an IEEE 802.11be WLAN and propose solutions applicable to both STR and non-STR MLDs. First, we describe the Sync scheme of an MLO and its design variants including Sync-FT. Next, we demonstrate the backoff count overflow problem of backoff compensation for Sync-FT and identify that the problem is aggravated once triggered due to continuing free-riding transmissions and compensations. Subsequently, we propose four solutions to mitigate this problem: limiting consecutive free-riding transmissions, limiting a compensated backoff value, using the contention window (CW) value of a main link, and balancing transmissions between links. Through comparative evaluation and analyses for dense single-spot and 3GPP indoor random deployment scenarios, we demonstrate in terms of throughput and latency that the proposed solutions successfully mitigate the backoff count overflow problem. We also investigate the coexistence performance of the solutions and demonstrate that the proposed solutions preserve coexistence performance with other MLDs and legacy SLDs while solving the backoff count overflow problem.

In summary, the main contributions of our work are listed as follows:

- Identification of the backoff count overflow problem of the Sync-FT channel access scheme of EHT with backoff count compensation;
- Design of potential solutions to mitigate the problem with minimal modification of standards;
- Comprehensive simulation work to show the performance gain of the proposed solutions with coexistence of multiple networks with different channel access mechanisms and legacy devices.

The rest of the paper is organized as follows. Section 2 explains the multilink Sync operation and Sync-FT modification, and its coexistence issue and a backoff compensation solution are presented in Section 3. Section 4 describes the backoff count overflow problem. Section 5 details the proposed solutions, and Section 6 evaluates them via simulation compared with other channel access schemes in coexistence scenarios. Finally, Section 7 concludes the paper.

## 2. Synchronous Multilink Channel Access

Sync is one of the multilink channel access mechanisms for EHT that has been considered to be used for transmissions of non-STR MLD. This operation uses both links in the backoff process, but a transmission is triggered only if the backoff counters of both links are zero. Once a link finishes its BO, it waits until the other link finishes BO. If another device occupies the channel while an MLD is in the waiting mode, the backoff count of the waiting link should be rechosen, and its BO is initiated with the new count. This means that a multilink transmission aligns the transmission start time in each link; thus, RF power leakage does not affect channel access and transmission. However, the requirement of the availability of both links at the same time leads to severe performance degradation especially when the channels are crowded. The inability to perform independent transmission in individual links may cause an MLD to suffer from waiting. Therefore, the use of Sync may not be able to exploit the potential of MLO for throughput enhancement.

In what follows, we describe two design variants of Sync.

### 2.1. Synchronous Operation with a Primary Link

The Sync with a primary link scheme (Sync-PL) is similar to the conventional wideband operation of IEEE 802.11 [4,5] In this scheme, an MLD runs a single BO in a primary link (PL) only and does not run in other links. When the primary link is about to reach a zero backoff count, the MLD performs a short clear channel assessment (CCA) for a point coordination function interframe space (PIFS) on each of the other links to check each’s availability. The links that are clear to send (idle) in a given short CCA period will then be able to send simultaneously with the primary link. The links in which the MLD transmits frames are the primary link and the other idle link(s). The MLD starts transmission in these links at the same time and ends transmission at the same time as well to avoid RF power leakage between the links. If all other links are found to be busy, the MLD transmits in the primary link only. Since only the primary link performs BO, a single link transmission is available only in the primary link. Sync-PL is expected to have better throughput performance than the basic Sync since at least a single link transmission is enabled. One concern is that a transmission is triggered only by the backoff completion of the primary link; thus, transmission opportunities highly depend on the load condition of the corresponding channel.

### 2.2. Synchronous Operation for Faster Transmission

Another enhancement of Sync is called Sync for faster transmission (Sync-FT) and has proven to be better in throughput performance than Sync. Sync-FT is a hybrid of Sync-PL and Async. As in Async, it lets the links of an MLD run individual backoff processes. When a link finishes its BO earlier than the others, the MLD performs a short CCA in each of the other links and starts a multilink transmission in a set of links sensed idle as in Sync-PL while freezing the backoff counts of the links. Once the links finish transmission, they resume individual backoff processes. The links that completed backoff at the time of transmission choose a new backoff count and initiate a new backoff process, while the other links resume BO with their remaining counts. We call the transmission of a link with BO completion as a main transmission and the transmission of a link before BO completion as a free-riding transmission. This operation enhances the multilink utilization of non-STR MLDs by avoiding the failure of link utilization due to RF power leakage. The operation is applicable to both STR and non-STR MLDs. Sync-FT enables both single-link and multilink transmissions in all links.

## 3. Coexistence Issue of Sync-FT and Backoff Count Compensation Solution

In this section, we describe the coexistence issue of Sync-FT and the backoff compensation solution.

### 3.1. Coexistence Issue

There may coexist legacy SLDs and EHT MLDs with different channel access schemes in networks. Therefore, the main coexistence scenarios are given below:

- MLD vs. legacy SLD;
- MLD vs. MLD with different channel access schemes.

Suppose that a legacy device starts a backoff process with the same backoff count as an MLD with Sync-FT. However, while the legacy station obtains a single transmission opportunity (TXOP), the MLD may obtain more TXOPs. This is because Sync-FT enables the MLD to transmit in a link before the backoff completion of the link. Such behavior prioritizes the channel access of the MLD and gives more TXOPs to it, resulting in long and frequent waiting times of the legacy device. When two MLDs, one with Sync-PL (MLD1) and the other with Sync-FT (MLD2) coexist, MLD2 is likely to obtain more TXOPs than MLD1 due to the aggressiveness of Sync-FT against Sync-PL.

In the basic design of Sync-FT, a link performing transmission by another link’s BO completion, which we call a free-riding link, resumes its ongoing backoff process after finishing transmission, with the remaining count value. This behavior makes the links under Sync-FT transmit more frequently than others that have to complete BO before transmission (e.g., legacy SLDs). In other words, this behavior makes the free-riding link go through a relatively deflated backoff count on average.

### 3.2. Sync-FT with Backoff Compensation

One of the proposed designs to tackle the coexistence issue of Sync-FT is having backoff count compensation after free-riding of a link [3]. This is a straightforward modification where the backoff count of a free-riding link is compensated for by an appropriate amount such that it finally goes through the backoff time given by its backoff counts on average. Before the additional backoff count value is added to the current count by compensation, a new backoff count should first be chosen. After a free-riding transmission finishes, the link stores the current backoff count value and rechooses a new backoff count. Then, it performs a new backoff process with an initial count as the sum of the stored backoff count and the rechosen one. This solution is simple in implementation, since it reuses existing functions of the backoff mechanism and can resolve the coexistence issue caused by Sync-FT’s aggressive behavior.

## 4. Backoff Count Overflow Problem

The backoff compensation solution described in the previous section leads to another problem called a backoff count overflow problem. This problem is that a link has a very large value of its backoff count, thus preventing it from finishing its countdown and transmission. In general WLANs, a greater backoff value is usually caused by consecutive transmission failures, causing a CW value to rise and in turn increasing a random backoff value chosen within the CW. This backoff count overflow problem, however, happens in Sync-FT with backoff compensation implementation even without consecutive transmission failures.

**Figure 1:**
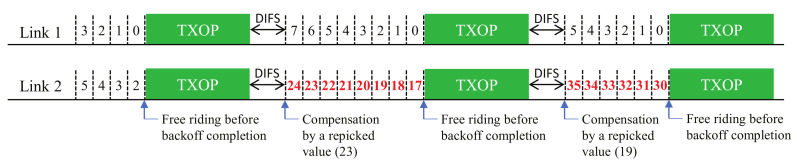
Illustration of the backoff count overflow problem (backoff count values after compensation are colored red).

The problem is illustrated in Figure 1. Upon completion of each free-riding transmission, the link compensates for its backoff count value by the remaining amount of the previous value. There is a possibility that one link may become stuck in consecutive free-riding transmissions and keep compensating its backoff count value, thus making the count increase indefinitely. This phenomenon happens especially when the channels of the links have heterogeneous load conditions; hence, some links in low load conditions keep decreasing their backoff values and make the other links keep free-riding with no chance of transmitting via their own BO completion. This is the case when links tend to have different backoff stages (i.e., different CW values) and thus heterogeneous backoff counts. As a free-riding transmission makes a link rechoose a backoff count from its current CW value (without a change in its backoff stage), a link with a higher backoff stage will statistically choose a larger backoff count value and, thus, have a higher chance of free-riding again. In other words, once a link performs a free-riding transmission, it will retain half of the current backoff count value on average (assuming that the BO completions of links are independent from each other and, thus, randomized between them) and add a new rechosen value, which is highly likely to make the resulting backoff count larger than the other links; If this happens consecutively, the link will always be a free-riding link.

**Figure 2:**
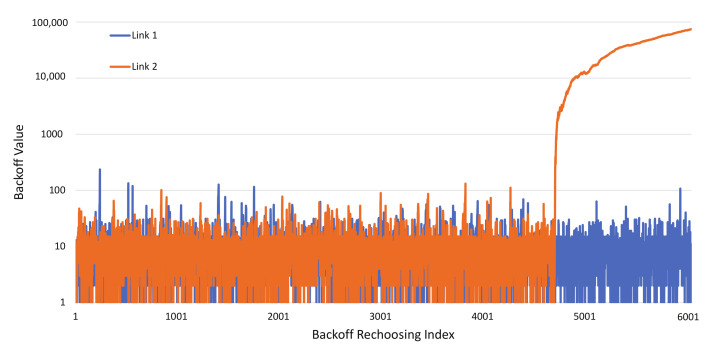
Backoff count evolution example for two links of an MLD.

Figure 2 shows the time evolution of the backoff count values of the two links of an MLD. In the figure, Link 1 and 2 have similar backoff count ranges in the beginning, but Link 2 suddenly keeps increasing its backoff count, while Link 2 still has a similar backoff count range. This is due to consecutive free-riding transmissions of Link 2. As mentioned above, a free-riding transmission of a link inflates the link’s backoff count when backoff count compensation is applied, which in turn increases the possibility of the link’s recurrent free-riding transmissions. This happens even when links are in the same load condition, but it is aggravated when they are in different load conditions. In such a condition, the backoff stages of links may be different from each other because of different occurrence rates of collisions.

## 5. Solutions to Backoff Count Overflow Problem

**Figure 3:**
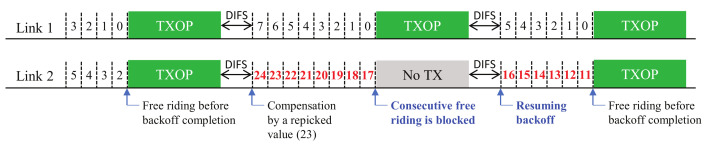
Proposal 1: limiting consecutive free-riding transmissions (backoff count values after compensation are colored red).

**Figure 4:**
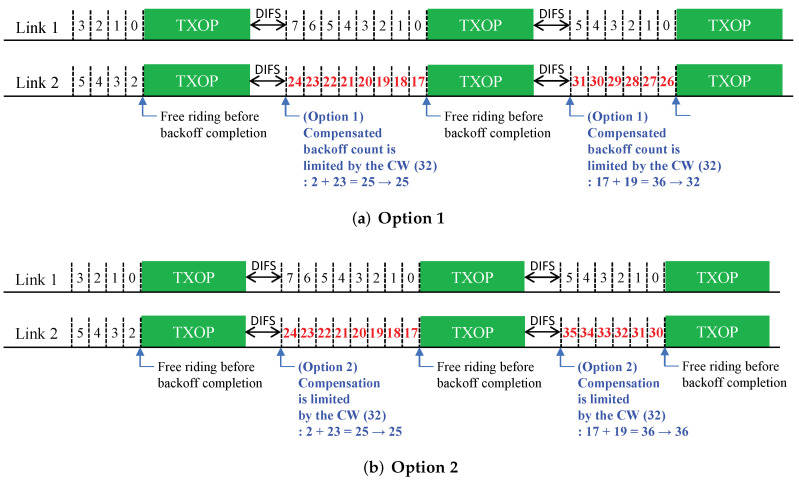
Proposal 2: limiting a compensated backoff value.

**Figure 5:**
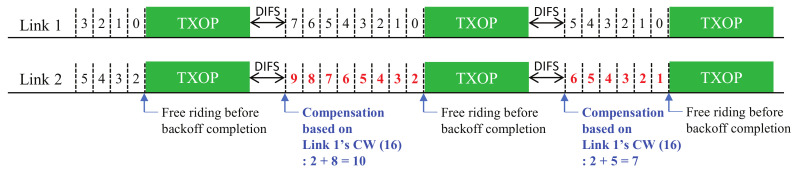
Proposal 3: using the CW value of a main link (backoff count values after compensation are colored red).

**Figure 6:**
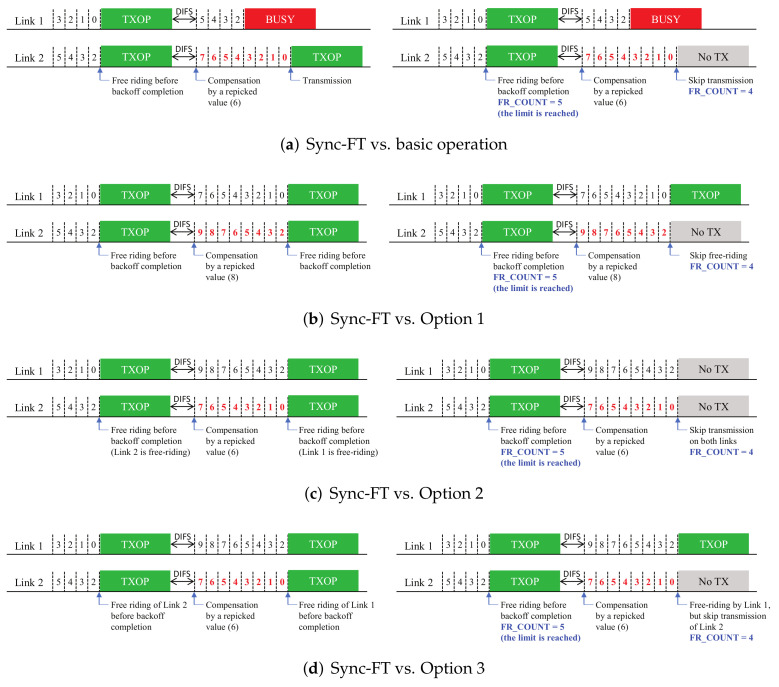
Proposal 4: balancing transmissions between links (backoff count values after compensation are colored red).

In this section, we describe the proposed solutions with additional design variants. Figure 3, Figure 4 and Figure 5 (Proposals 1, 2, and 3, respectively) illustrate the changed operation of Link 2 against the Sync-FT operation with compensation illustrated in Figure 1 (the operation of Link 1 remains the same as Figure 1, since only Link 2 performs free-riding) while Figure 6 (Proposal 4) illustrates another example operation of Sync-FT (with backoff compensation) and the changed operation side by side. We assume in the figures that Link 1 is in the first backoff stage (CW = 16) and Link 2 is in the second backoff stage (CW = 32).

### 5.1. Proposal 1: Limiting Consecutive Free-Riding Transmissions

The main cause of an indefinite increase of a backoff count value, resulting in a backoff overflow, is consecutive free-riding transmissions of a link. This solution is made to alleviate the problem by limiting the number of allowed consecutive free-riding transmissions of each link. As illustrated in Figure 3, if a link performs a free-riding transmission, it starts its consecutive counter with value one. The next free-riding transmission right after the first one will result in an increment of the consecutive counter by one. The counter will keep increasing through consecutive free-riding transmissions of the link. Once the counter reaches its limit, the link will block its next free-riding transmission and reset the counter to zero. The counter will also be reset if there is a transmission caused by the link’s own BO completion. The blocked free-riding transmission will keep its remaining backoff count value and continue to decrease the value after a main transmission finishes without a compensation. Setting the limit to one is the same as disabling any consecutive free-riding transmission, i.e., one link can free-ride only once, then must finish its own backoff process to transmit.

### 5.2. Proposal 2: Limiting a Compensated Backoff Value

A backoff overflow is caused by adding compensation to what is already a large backoff count value. In order to prevent that from happening, we designed the second solution to limit the compensated backoff count value itself. There are two ways of limiting the compensated backoff count value:

- (Option 1) limiting the total backoff count: The compensated backoff count, which is the sum of a rechosen number and a compensation value, is limited by a certain value (e.g., current CW value or that multiplied by a certain factor). It is illustrated in Figure 4.
- (Option 2) limiting a compensation value: Instead of limiting the total backoff value, only the compensation value to be added to the new rechosen number is limited. This is to prevent adding an overlarge value to a large remaining backoff count.

In both solutions, we can ensure the new backoff value remains at a smaller value and increase the chance of a free-riding link to complete its backoff process the next time.

### 5.3. Proposal 3: Using the CW Value of a Main Link

As explained previously, the backoff overflow problem is aggravated if the links of an MLD have different backoff stages. The case is especially true when a free-riding link has a higher backoff stage than the link of the main transmission, because this can cause the randomly chosen count value of the free-riding link to become higher. In the solution illustrated in Figure 5, a free-riding link will use the same CW value as that of the link performing a main transmission, without considering its own backoff stage or current CW value. By using the same CW value as the main link, the free-riding link will have a higher probability to finish its own backoff process at a similar time as the main link. The same CW value with the main link will only apply to the new compensated rechosen value and will not affect transmissions by other means.

### 5.4. Proposal 4: Balancing Transmissions between Links

The main idea of this solution is to balance the number of TXOPs between links so that equal opportunities are given between them. The solution introduces a FR_COUNT value. This is a variable that increases when a link performs a free-riding transmission and decreases when it skips a transmission caused by its own BO completion, but it can never be lower than zero. Skipped transmissions will be excluded from compensation, thus mitigating the backoff overflow problem. The solution has several optional features to be implemented according to what to skip when the FR_COUNT of a link becomes higher than a limit as illustrated in Figure 6:

- Basic: A single link transmission of the link is skipped when its backoff count becomes zero.
- Option 1: A free-riding transmission is skipped when another link finishes BO.
- Option 2: Both main and free-riding transmissions are skipped together when the link finishes BO.
- Option 3: Only a main transmission is skipped while a free-riding transmission of another link is still allowed when the link finishes BO.

## 6. Performance Evaluation

We evaluated and compared the performance of MLO with Sync-FT with the proposed solutions. Both throughput and latency performance were observed in the evaluation. The coexistence of MLDs with legacy SLDs was also considered. In addition, backoff count values were observed as a way to determine the effectiveness of the proposed solutions to the backoff overflow problem.

### 6.1. Environmental Setup

Each MLD had two links (Link 1 and Link 2) working in individual channels. RF power leakage between the links of an MLD was considered, so all MLDs were non-STR MLDs. Each EHT basic service set (BSS) was composed of an access point (AP) and connected device(s) (a single device in a single-spot scenario and multiple devices in a 3GPP indoor scenario). APs and MLDs were equipped with MLO capability, while legacy devices were not. All devices used the modulation and coding scheme (MCS) 7 in 80 MHz (i.e., data bit rate of 680.6 Mbps) with the aggregation MAC service data unit (AMPDU) of 64 MPDUs, where each MPDU was 1500 bytes long. We considered full-buffer traffic conditions. Proposal 1 used one as a consecutive free-riding transmission limit, Proposal 2 used Option 1, and Proposal 4 used Option 1 with the FR_COUNT limit set to five. The operation frequency band of the network was 5 GHz, and the channel bandwidth was 20 MHz. Other system parameters followed those for the simulation scenario [6] and evaluation methodology [7] used for IEEE 802.11ax as listed in Table 1. All simulation results were obtained by averaging over five runs; in each run, 50 s were simulated.

**Figure 7:**
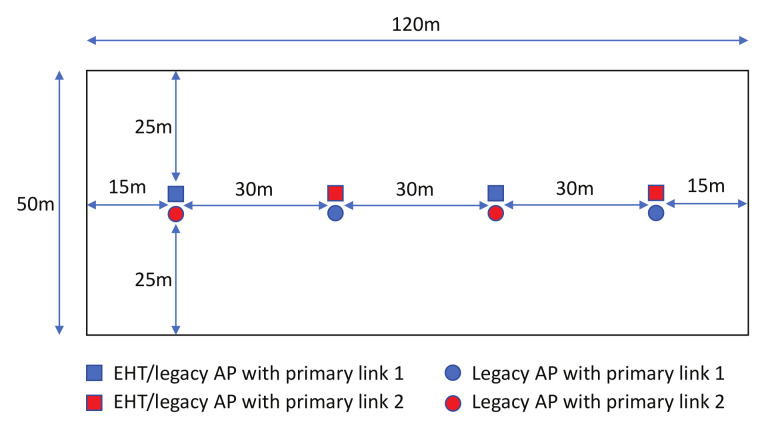
3GPP indoor deployment scenario.

We considered two deployment scenarios:

- *Single-spot deployment*: We placed all BSSs in one spot with no distance between them. Overlapping BSSs (OBSSs) were added in each of the links at the same time, i.e., one OBSS means one legacy BSS in either channel of Link 1 and Link 2.
- *3GPP indoor deployment* [8]: We followed Figure 7 to deploy the BSSs in the field. Each BSS consisted of 20 connected devices working in the respective link mentioned in the figure. The devices were randomly placed within their serving AP’s coverage area.

Each deployment scenario had two coexistence scenarios:

- *MLD vs. MLD*: We considered EHT BSSs, each with its own multilink channel access scheme. One half of EHT BSSs was set using Sync-PL, and the rest used various channel access schemes, including the proposed solutions. The goal of this scenario was to examine the performance of the proposed solutions compared to the Sync-PL channel access scheme.
- *MLD vs. Legacy SLD*: We considered two legacy BSSs in each of the channels per 1 EHT BSS. The EHT BSS used various channel access schemes, including the proposed designs. The goal was to examine the performance of legacy BSSs, which is affected by the EHT BSSs.

### 6.2. Single-Spot Deployment

#### 6.2.1. MLD vs. MLD

**Figure 8:**
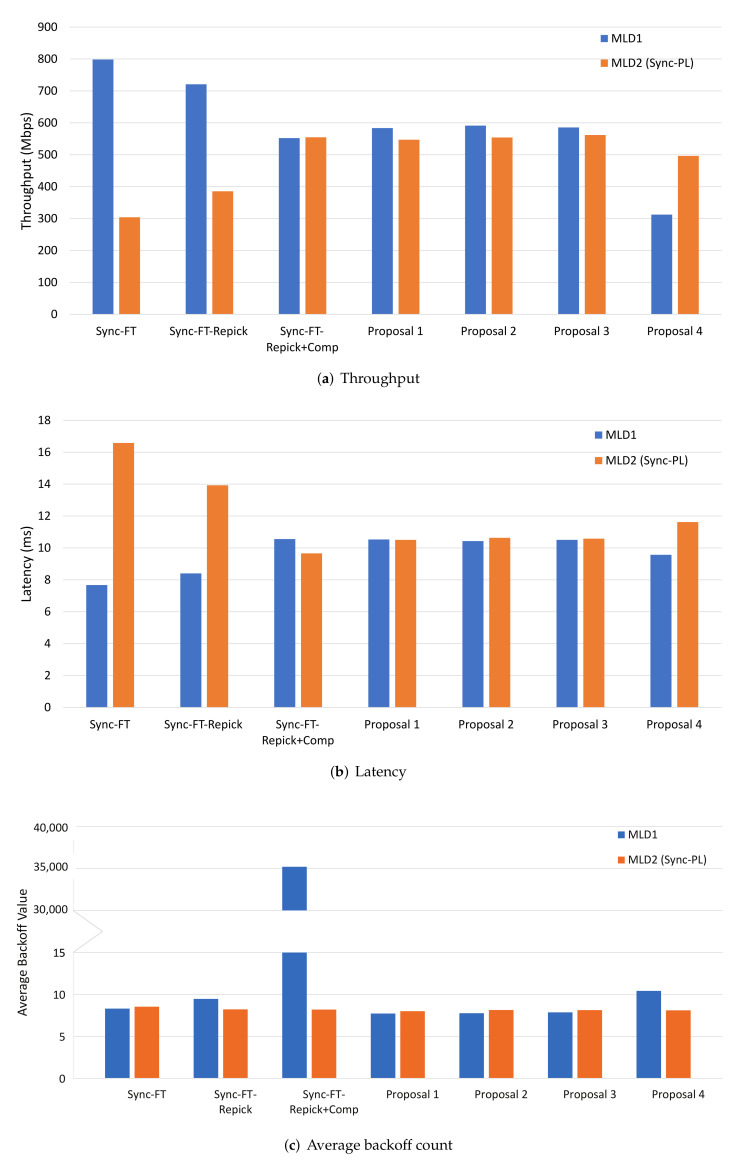
MLD vs. MLD performance in the single-spot deployment scenario.

Figure 8 shows the performance results with one MLD BSS with Sync-PL and the other with various channel access schemes. Sync-PL was chosen as the benchmark, because it suits a non-STR MLD better than the basic Sync operation. In the figure, along with the proposed solutions, we also considered Sync-FT (with no backoff compensation), Sync-FT with a rechoose option (Sync-FT-Repick), and with backoff compensation (Sync-FT-Repick+Comp). From the throughput and latency results, we see that Sync-FT and its modified rechoose design (MLD1) had a significantly higher performance than Sync-PL, while the other schemes (except Proposal 4) showed only slightly better performance than Sync-PL. This is because the earlier designs are highly aggressive against Sync-PL. On the contrary, the proposed solutions (except Proposal 4) coexisted better with Sync-PL. One point to note is that Proposal 4 had a worse performance than Sync-PL, which was caused by the excessive penalties given to the free-riding link. Figure 8c shows that our solutions alleviated the backoff overflow problem. The problem was severe, with Sync-FT-Repick+Comp showing the largest value of the average backoff count. Sync-FT and Sync-FT-Repick did not show the problem since they did not use backoff compensation and, thus, showed aggressive behavior. The proposed solutions achieved both harmonized coexistence and alleviation of backoff overflow.

#### 6.2.2. MLD vs. Legacy SLD

**Figure 9:**
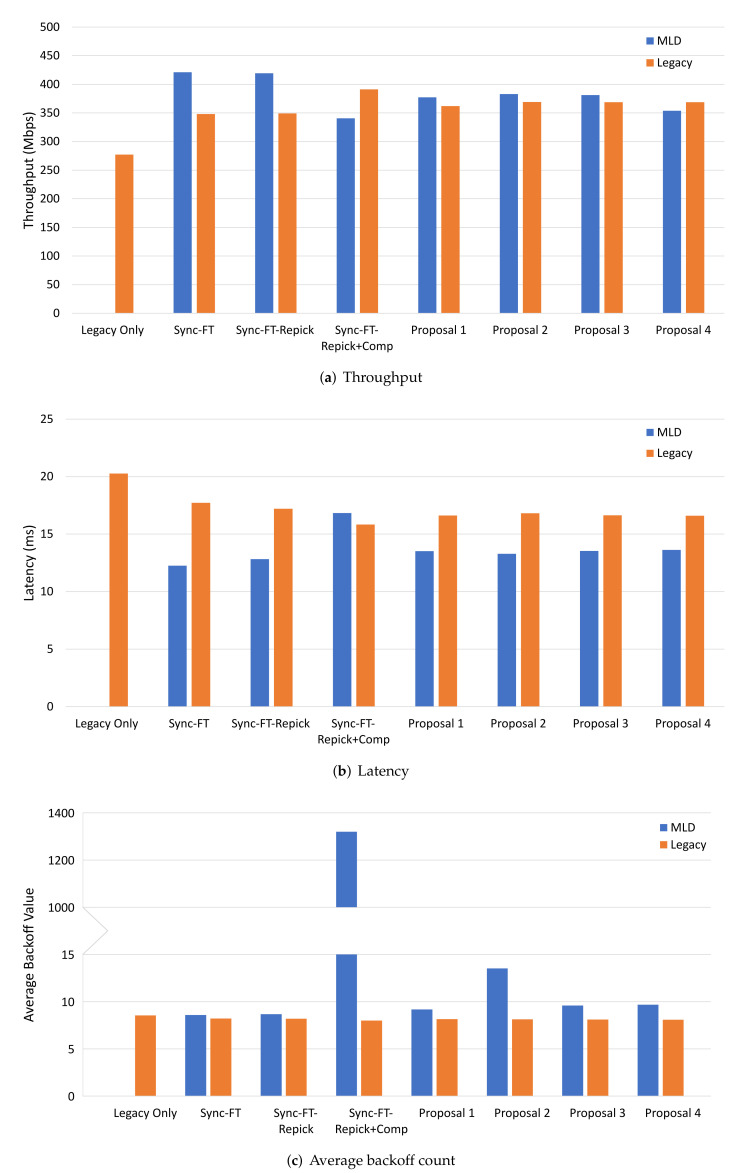
MLD vs. legacy SLD performance in the single-spot deployment scenario.

The results of this scenario are presented in Figure 9. The case of legacy BSSs only (with no MLD BSS) is shown first labeled as “Legacy Only” in the graphs as a comparative measure to examine whether an EHT BSS has a positive or negative effect on the legacy performance. Compared to Sync-FT and Sync-FT-Repick, Sync-FT-Repick+Comp had a lower MLD performance in throughput and latency. This can be considered as due to the conservative behavior of Sync-FT-Repick+Comp; however, it is due to the backoff overflow problem as shown in Figure 9c. All the proposed solutions succeeded in suppressing the Sync-FT’s channel access aggressiveness, while still maintaining good performance. Their average backoff count values were well limited compared to Sync-FT-Repick+Comp. The performance enhancement by mitigating the problem was especially noticeable in terms of latency; Figure 9b shows that Proposal 2 had 21.5% lower latency than Sync-FT-Repick+Comp. This implies that the proposed solutions were effective in solving the backoff overflow problem, while supporting the coexistence with legacy SLDs. On the other hand, it is also shown that the performance of legacy BSSs was also higher when half of them were replaced with an EHT BSS. This is due to the higher effectiveness of MLO in utilizing the channel medium and, thus, more room given to remaining legacy BSSs for channel access.

### 6.3. 3GPP Indoor Deployment

The indoor path loss model of the IEEE 802.11ax simulation scenario was used as given below:

(1) $$\begin{array}{c} PL\left(d\right)=40.05+20\log_{10}\left(f_{c}/2.4\right)+35\log_{10}\left(d/5\right) \end{array}$$

where *d* is a transmitter–receiver distance in meters, and $f_{c}$ is the center frequency in GHz. Then, the receive power ($P_{r}$) is obtained as

(2) $$\begin{array}{c} P_{r}=P_{t}+A_{t}-PL\left(d\right)+A_{r} \end{array}$$

where $P_{t}$ is the transmit power, and $A_{t}$ and $A_{r}$ are the antenna gains of the transmitter and receiver, respectively. The noise figure is 7 dB, and the noise floor is −94 dBm. We considered the transmission bit rates of IEEE 802.11ac (6.5 to 78 Mbps). The signal–to–noise ratio (SNR) vs. packet error rate (PER) curves of the IEEE 802.11ax’s evaluation methodology [7] were used for packet error generation. For link adaptation, the highest bit rate with a PER lower than 10% was selected [9,10]. The control frames were transmitted at the lowest bit rate.

#### 6.3.1. MLD vs. MLD

**Figure 10:**
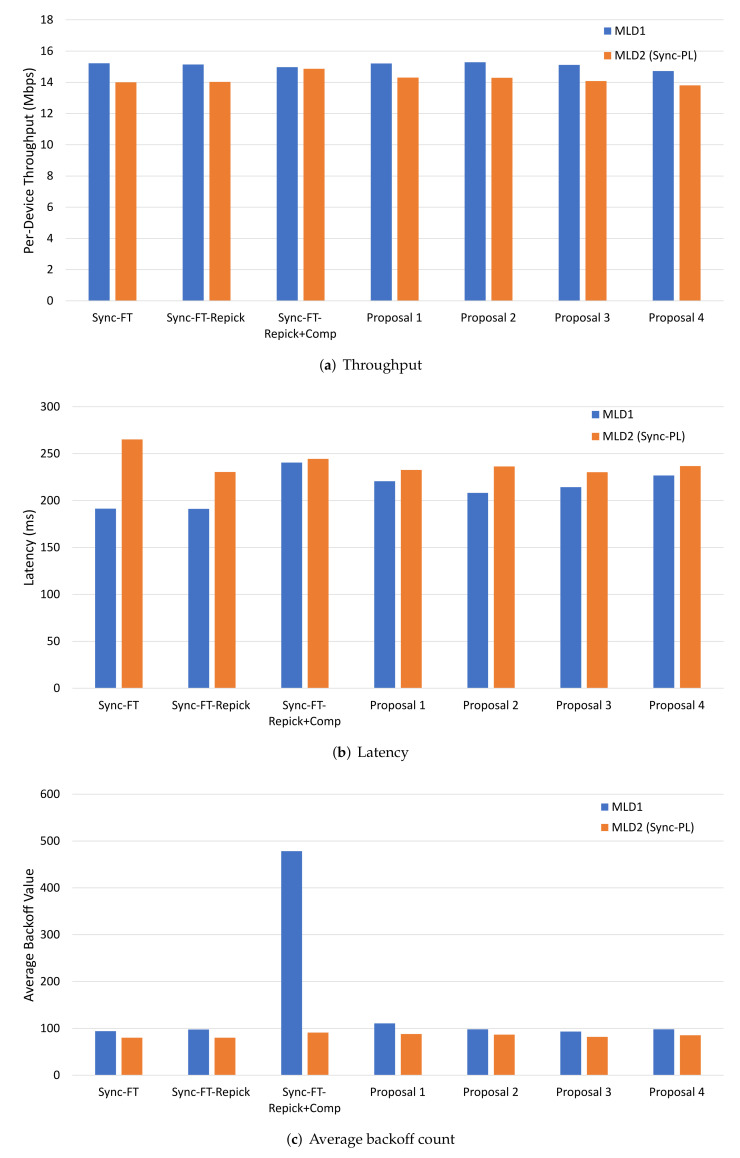
MLD vs. MLD performance in the 3GPP indoor deployment scenario.

With the same reasoning as the single-spot deployment, Sync-PL was also used as a comparative benchmark in this scenario. The evaluation results are given in Figure 10. From both throughput and latency results, we can see that Sync-FT-Repick+Comp had the worst performance compared to Sync-PL because of the backoff overflow problem. All of the proposed solutions showed increased performance that was similar to Sync-FT and Sync-FT-Repick. This means that they alleviated the backoff overflow problem. Another proof that the proposed solutions effectively alleviated the problem is shown in Figure 10c. The problem happened with Sync-FT-Repick+Comp, showing a significantly large value of the average backoff count. The problem was considerably alleviated by the proposed solutions, as they showed small average backoff count values. Among the solutions, Proposal 2 (limiting a compensated backoff value) showed a relatively high backoff count value, meaning it was less effective than the other solutions.

#### 6.3.2. MLD vs. Legacy SLD

**Figure 11:**
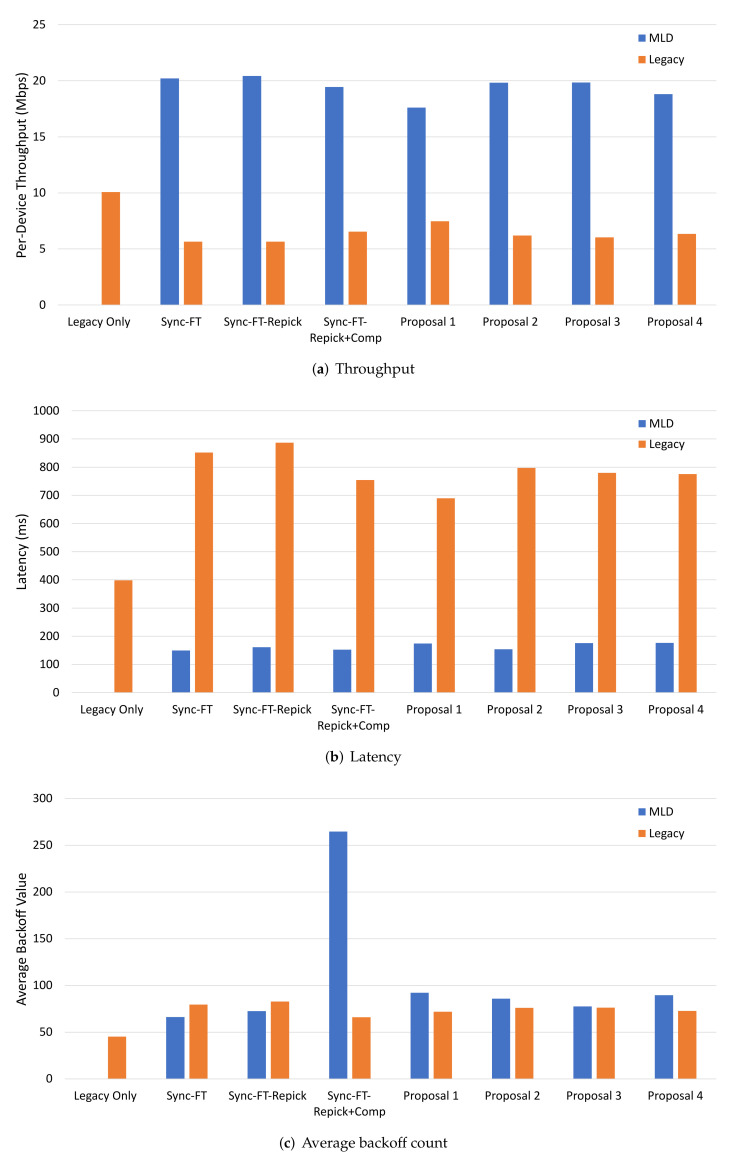
MLD vs. legacy SLD performance in the 3GPP indoor deployment scenario.

The evaluation results of this scenario are presented in Figure 11. In the figure, Sync-FT-Repick+Comp showed lower performance than Sync-FT and Sync-FT-Repick, especially in the latency performance, which was possibly caused by backoff overflow. Figure 11c shows that Sync-FT-Repick+Comp suffered the backoff overflow problem. All proposed solutions mitigated the problem without ruining coexistence with the legacy BSSs. Proposals 1 and 4 showed slightly worse MLD performance, which was more noticeable in the latency performance. It is shown in Figure 11c that the average backoff count values of all proposed solutions were as small as Sync-FT and Sync-FT-Repick, thus proving that they were effective in solving the backoff overflow problem. As observed in the results of the single-spot deployment scenario, the performance enhancement in this deployment scenario was also noticeable in terms of latency; Figure 11b shows that Proposal 2 had 13.5% lower latency than Sync-FT-Repick+Comp.

## 7. Conclusions

Sync-FT is one of the best options for multilink channel access in IEEE 802.11be WLANs, especially for non-STR MLDs. Due to the raised coexistence issue of Sync-FT resulting from its aggressive channel access behavior, the backoff count compensation solution can be applied to it. In this paper, we identified the backoff count overflow problem of Sync-FT with backoff count compensation, which happens due to continuing free-riding transmissions and backoff count compensations, thus causing an unlimited increase in the backoff count value. We proposed four solutions to alleviate the problem and showed through comprehensive evaluation that all proposed solutions were effective in mitigating the problem while still preserving coexistence with other MLDs and legacy SLDs.

## Figures and Tables

**Table 1:** Simulation parameters.

Parameter	Value
Number of links per MLD	2
Channel bandwidth of a link	80 MHz
Number of STAs per BSS	1
MCS	7 (680.6 Mbps)
Traffic generation	Full buffer
Max aggregation size	64 MPDUs
MPDU size	1500 bytes
Slot length	9 us
SIFS	16 us
DIFS	34 us
CWmin	16
CWmax	1024
Center frequency	5 GHz
STA transmit power	18 dBm
AP transmit power	21 dBm
STA antenna gain	−4 dBi
AP antenna gain	+2 dBi
Noise figure	7 dB

## Data Availability

Not applicable.

## Abbreviations

The following abbreviations are used in this manuscript:

AP	Access Point
ASync	Asynchronous channel access operation
BO	BackOff procedure
BSS	Basic Service Set (BSS)
CCA	Clear Channel Assessment
CW	Contention Window
CWmax	Contention Window maximum
CWmin	Contention Window minimum
DIFS	Distributed coordination function InterFrame Space
EHT	Extremely High Throughput
FT	Faster Transmission
MAC	Medium Access Control
MCS	Modulation and Coding Scheme
MLO	MultiLink Operation
MLD	MultiLink Device
OBSS	Overlapping Basic Service Set
PIFS	Point coordination function InterFrame Space
RF	Radio Frequency
STR	Simultaneous Transmission and Reception
SLD	Single-Link Device
SLO	Single-Link Operation
Sync	Synchronous channel access operation
WLAN	Wireless Local Area Network
